# Monoclonal antibody therapy for prevention of severe disease in nosocomial COVID-19

**DOI:** 10.1017/ash.2022.123

**Published:** 2022-05-16

**Authors:** Adam Zimilover, Thien-Ly Doan, Sumeet Jain, Prashant Malhotra

## Abstract

**Background:** In November 2020, the FDA issued an emergency use authorization (EUA) for monoclonal antibodies (mAbs) to be used in outpatients with COVID-19 infections who are at a high risk of progressing to severe disease. However, becuase the EUA had limited indications for inpatients, data on their use in hospitalized patients are limited. In this study, we have described the use of mAbs among hospitalized patients with nosocomial COVID-19. **Methods:** We retrospectively analyzed cases of nosocomial COVID-19 in 2 tertiary-care hospitals from November 1, 2020, to October 11, 2021, and we identified patients who received mAbs. The study period was prior to the ο (omicron) variant (B.1.1.529) being detected in the United States, and infections in the patients were likely primarily with the α alpha variant (B.1.1.7) and the δ (delta) variant (B.1.617.2) of SARS-CoV-2, which responded well to treatment with bamlanivimab and casirivimab–imdevimab. All patients had a negative SARS-CoV-2 PCR on admission. Data on clinical outcomes, including administration of medications for COVID-19, increases in oxygen requirements, ICU admission, mechanical ventilation, and death were collected by a review of the electronic medical record. The study was approved by the institutional review board with expedited approval. Descriptive statistics, such as means and standard deviations of continuous variables and proportions of categorical events or variables, were tabulated to describe patient characteristics and outcomes. **Results:** The 71 patients included in the study (age range, 39–89 years; median age, 70 years; 51% female) received either bamlanivimab (n = 31) or casirivimab–imdevimab (n = 40). The length of stay ranged from 6 to 242 days (median, 26 days). The comorbidities present included cardiovascular disease (56%), diabetes (45%), obesity (31%), autoimmune disease or immunosuppression (27%), kidney disease (23%), and pulmonary disease (20%). Most of the patients included in the study were incompletely vaccinated or unvaccinated (94%) and were negative for SARS-CoV-2 antibodies (81%). Prior to receiving the mAbs, 23% of patients required supplemental oxygen, including 3 patients who required mechanical ventilation. These patients required oxygen support due to non–COVID-19–related conditions. After mAb infusion, 72% of patients had no increase in their oxygen requirements, and 93% did not progress to mechanical ventilation. Overall, 7 deaths were attributed to COVID-19 among the studied patients (10%). **Conclusions:** Our study describes the use of mAbs in hospitalized patients with nosocomial COVID-19. Most of the patients who received mAbs had no progression to severe COVID-19, despite having significant comorbidities. The use of mAbs in nosocomial COVID-19 may be associated with beneficial outcomes.

**Funding:** None

**Disclosures:** None

